# The cyber-enhanced tank: a novel concept for increased realism and multi-modal monitoring in tank based finfish aquaculture research

**DOI:** 10.3389/frobt.2025.1629884

**Published:** 2025-11-12

**Authors:** Dimitris Voskakis, Martin Føre, Eirik Svendsen, Aleksander Perlic Liland, Sonia Rey Planellas, Harkaitz Eguiraun, Pascal Klebert

**Affiliations:** 1 Department of Engineering Cybernetics, Faculty of Information Technology and Electrical Engineering, Norwegian University of Science and Technology, Trondheim, Norway; 2 SINTEF Ocean AS, Trondheim, Norway; 3 Institute of Aquaculture, School of Natural Sciences, University of Stirling, Scotland, United Kingdom; 4 Department of Graphic Design and Engineering Projects, Faculty of Engineering of Bilbao, University of the Basque Country UPV/EHU, Bilbao, Spain; 5 Research Centre for Experimental Marine Biology and Biotechnology, University of the Basque Country PiE-UPV/EHU, Plentzia, Spain

**Keywords:** aquaculture, cyber-enhanced tank, fish monitoring, precision fish farming (PFF), event camera, acoustic sonar

## Abstract

In recent years, several studies that analyze and interpret fish behavioral patterns in aquaculture settings have been published. Understanding how the fish react and respond to various scenarios and treatments can help provide insight and knowledge on how to achieve sustainable and efficient aquaculture production. Many of these research efforts have been conducted in land based tanks as this allows for closer and more continuous monitoring of the fish than what is possible at commercial facilities, essentially improving data quality and hence the possible insights to gain from these. However, most experimental tanks are closed-loop environments that are not particularly similar to commercial production units, as a consequence the results obtained in these systems are not directly transferable to industrial setups. Moreover, tank monitoring in such trials is often done using a single or a limited selection of different observation modes, which may not be sufficient to capture the full dynamics of fish responses. The present study seeks to address these challenges by developing the Cyber-Enhanced tank environment for aquaculture research. This concept features a tank environment setup to simulate the prevailing conditions in aquaculture units, mimicking natural light conditions, hiding sensors and other systems to reduce impacts on the fish and potential collisions, and using a tank color known to stimulate positive welfare in farmed fish. The tank was equipped with a novel sensor suite for high-fidelity detection and monitoring of fish behaviors based on a combination of an event camera, a scanning imaging sonar and conventional cameras. This innovative concept represents a step towards conducting experimental setups that are both more realistic in that conditions resemble those in commercial facilities and that uses a multi-modal sensor approach to capture details in fish responses in behaviors. The setup will be used as a basis for future fish responses experiments monitoring experiments in intensive aquaculture tanks.

## 1 Introduction

### 1.1 Commercial fish farming

For thousands of years, aquatic resources have been a vital source of food for communities in coastal and marine areas. Several studies have highlighted the importance of this precious food in their daily lives. The first examples of humans exploiting marine resources, in various forms such as fishing, aquaponics and aquaculture occurred in the ancient civilizations of Hellas and China.

Production growth in aquaculture has been remarkable over the recent decades, with global production increasing from 2.6 million metric tons (mt) in 1970 to 87.5 million mt in 2020 (FAO, 2024). Intensive production of carnivorous finfish has developed into one of the most prominent sectors within aquaculture, largely because of the high nutritional value of the end product and its commercial appeal, and because recent advancements in farming methods have rendered this production form efficient and affordable.

The salmon industry has been considered a particularly successful segment in this sector, and has enjoyed rapid growth much due to significant research advancements and innovations within production forms and technology for salmon production in Norway (Afewerki et al., 2023).

There exist a plethora of different systems for the commercial production of Atlantic salmon. The most common practice by far is farming in marine fish farms featuring net pens suspended from floating structures that in turn are moored to the seabed. To extend the volume of the net pens, and hence the volume available for the fish, their lower ends are typically attached to weights and sinker tubes. The porosity of the net wall makes the net pens open to the surrounding water such that crucial cage environment variables such as oxygen and temperature are directly provided from the ambient environment (Klebert et al., 2013). This has stimulated the development of many new innovations within salmon farming practices and methodologies designed to counteract such challenges (Barrett et al., 2020; Føre et al., 2022) resulting in several sustainable approaches and concepts using emerging technologies (Araujo et al., 2022). One such approach is closed production where the fish are kept in units that are closed to the environment, thereby reducing the exposure to environmental features that may be negative for fish welfare, sustainability and production capacity (Barrett et al., 2020). Closed production can either be realized in marine farms or land-based systems, and specially designed feeding and sensors systems for such units can provide a technological foundation to ensure suitable conditions for the fish. Another concept that is gaining interest is the movement of production to more exposed locations further from shore (Bjelland et al., 2025). Exposed farming operations are often based on open cages much like conventional farming methods, and are based on the assumption that the waters further from shore feature fewer parasites and pathogens than coastal archipelagos and fjords. While the main motivation behind the development of these new concepts is to avoid many of the challenges in conventional farming, they are also industrially important when considering the development of the finfish market, as it is unlikely that one or a few production forms alone will be sufficient to meet future demands.

Irrespective of the production format, industrial salmon farms feature large volumes containing high fish densities, and day to day farm management usually entails regular operations such as feeding, cleaning and farm maintenance, and otherwise seeking to avoid disturbing the fish. It is possible to do some experiments in such venues to for example, monitor fish responses to environmental conditions (Johansson et al., 2006), assess growth over time (Føre et al., 2016) or assessing physical phenomena like hydrodynamics (Lader et al., 2008; Winthereig-Rasmussen et al., 2016; Klebert et al., 2015).

However, commercial facilities are often not suitable arenas for preliminary studies targeting more detailed or individual based responses or features, since this often requires a tighter control of the experimental conditions than what is possible to achieve in such systems. This has resulted in that most aquaculture related experiments are conducted in experimental facilities specifically designed to allow closer control of the conditions and continuous monitoring of the fish. To fulfill these requirements, these facilities are often designed and run in ways that differ greatly from the commercial facilities. In addition, most research infrastructures are realized with land based tank systems mainly because the infrastructure (i.e., power, internet, water supply) required for experiments is much easier to get on land than at sea. Research units are also often smaller than industrial tanks and cages, previous studies have found that the scale of production units have an impact on the fish, even affecting their growth (Espmark et al., 2017). Finally, the production environment in these type of tanks is usually different from those fish will encounter at farms, especially in crucial factors such as lighting, flow fields and temperatures.

### 1.2 Natural light simulation

Based on the disparities between the conditions in commercial farms and those in a standard land based tank, it is apparent that researchers should develop setups that approximate the conditions on commercial farming sites as well as possible to achieve industrially relevant outputs. The most important measure towards ensuring this is to apply environmental enrichment. Enrichment in aquaculture is mainly used to provide the fish with stimuli that have positive impacts on fish welfare mainly to ensure improved animal welfare and more ethical production, but also because several studies have indicated the importance of fish welfare in providing optimal production environments and better practices (Aubin et al., 2009). Results from the introduction of enrichment measures are usually evaluated by interpreting fish behavior as observed by sensor systems in the production unit (Arechavala-Lopez et al., 2022). The enrichment process can also be used to provide a more realistic farming environment for the fish, thereby contributing to closing the gap between research facilities and farming sites.

A major topic within enrichment is the use of artificial illumination and several surveys have outlined the advantages of such practices regarding fish swimming behavior and growth. In a study conducted by one of the authors of this study and collaborators, artificial lights were used to control vertical fish movements and distribution patterns, offering insight into how fish can be steered through this stimuli (Føre et al., 2013; Wright et al., 2015; Herbert et al., 2011). In Wright et al. (2015), specially designed artificial lights were placed in aquaculture cages containing Atlantic salmon, the outcome of which was that the fish exhibited modified swimming patterns in response to lighting. Herbert et al. (2011) did a similar study, albeit in tanks, and found similar results. Together, these results imply that distribution and swimming depth may be possible to control using artificial lights, a feature that was further explored through mathematical simulations by Føre et al. (2013). In more recent studies, scientists have also investigated the swimming pattern and the growth maturation of the salmon in real aquaculture units, outlining the growth effect regarding external light sources (Hansen et al., 2017). Another enrichment measure relevant for fish welfare is to apply appropriate colors to the interior of the production unit, which is especially relevant for tank based trials. Earlier studies have explored the impacts of various color intensities on fish performance, and found different colors to elicit different responses (McLean et al., 2008). These results have later been reviewed, showcasing the importance of tank colors, and asserting that certain fish species perform better when subjected to specific colors (e.g., green, blue and black) than others (McLean, 2021). While many of the conditions at fish farms are hard to replicate in lab setups, the intelligent use of artificial lights and tank color can, in combination with camouflaging instruments and devices, render the visual tank environment perceived by the fish more similar to that in a commercial production unit. This could represent a first step towards achieving farm realistic conditions in experimental tank setups.

### 1.3 Fish behavior and welfare monitoring

Since fish welfare is not easy to observe directly, research aimed at welfare assessment and monitoring often need to target other observable variables that are believed to be linked with the welfare of the animal. Based on Norecopa (Norway’s National Consensus Platform), welfare is defined in terms of animal’s perception (intrinsic experience) of itself. Since it is impossible to observe welfare in such terms directly, it can only be evaluated by proxies, underscoring the importance of current study. Variables derived from behavior are often easier to monitor than those linked with physiology since behavioral expressions usually entail spatial movements that are externally detectable. Furthermore, fish behavior has in itself intrigued the scientific community, as it can result in important information about the interactions between species, different environmental changes, and external stress factors. In light of recent developments toward replacing manual labor with automation and technologies such as precision fish farming (Føre et al., 2018), there has been a drive towards adapting new technologies for more detailed, continuous and robust observation of fish behaviors in aquaculture. Several studies have thus aspired to observe fish behaviors in fish farming systems using different sensing principles systems (Cui et al., 2024; Burke et al., 2025). Methods exploiting remote sensing principles including vision based sensors such as cameras and other optical instruments (Saberioon et al., 2017) and hydroacoustic devices (Li et al., 2024a) are particularly interesting due to their non-invasiveness and ability to gain information on both individual fish and groups.

Vision based sensors have become the most commonly used technology for monitoring fish in aquaculture, and have thus had a leading role in leveraging technological solutions for more accurate fish management. Several studies have used waterproof cameras to estimate fish morphometric parameters (e.g., Shi et al., 2020; Voskakis et al., 2021; Chuang et al., 2015) and behavioral patterns (e.g., Georgopoulou et al., 2021; Eguiraun and Martinez, 2023; Burke et al., 2025). Other surveys have applied cameras after retrieving the fish from the aquatic environment to accurately measure various fish parameters such as fish length (Hsieh et al., 2011; Monkman et al., 2020). The advancement of optical sensor technology has also resulted in different types of camera applications and the development of innovative processing methodologies providing new insights (Pinkiewicz et al., 2011; Zhou et al., 2019).

While hydroacoustic technologies have mainly been used for underwater vehicle navigation, inspection and guidance (Skaldebø et al., 2024; Zerr et al., 2005; Hansen et al., 2003), emerging studies have highlighted the utilization of such technology in aquaculture settings (Li et al., 2024a). The most common application of sonars (single beam) or echo sounders has been to study the spatial distribution of fish (Tao et al., 2010; Boswell et al., 2007) often providing insights into specific patterns such as vertical distribution (Oppedal et al., 2007). Others have sought to study individual fish swimming behavior (Schwarz, 1985; Plimpton et al., 1997; Arrhenius et al., 2000) or individual sizes (Knudsen et al., 2004) using split-beam sonars placed in sea cages. More recent studies have sought to explore the potential of even more complex active hydroacoustic systems such as multibeam sonars in aquaculture, demonstrating the possibility of obtaining 3D spatial data (Kristmundsson et al., 2023).

### 1.4 Objectives

As emerging technological advancements are rapidly transforming many aspects of research and daily life, it is imperative to preserve the concept of natural development processes of living organisms. The present study introduces the cyber-enhanced tank (CET) which is a conceptual framework that bridges the gap between experimental tanks and commercial production facilities. To demonstrate the CET concept, we have expanded an existing physical tank setup with technological means including a novel adjustable submerged lighting system capable of simulating the conditions in an aquaculture farm unit and an innovative sensor suite for fish monitoring. This is an updated and expanded version of a previous research outcome (Voskakis et al., 2024) and offers an arena for conducting controlled experiments in a more realistic environment and which offers multiple non-invasive modes for observing fish behavior. Increased realism is sought achieved by designing the lighting system to emit a light spectrum resembling that of natural sunlight and to follow the natural photoperiod and that is not perceived as a point source by the fish. A further enrichment measure towards this end is to choose the tank wall color from a selection of colors previously identified as having positive impacts on fish welfare. The sensor suite is specifically designed for behavioral monitoring and targets several features of the fish behavior by simultaneously using four different sensors (i.e., a surveillance camera, stereo vision, an event camera and a scanning sonar). In addition to providing multi-modal observation, the sensor suite combines technologies that have seen little or no use as research tools in tank based experiments with more conventional observation methods, thereby enabling new methods for monitoring fish. These sensor systems are either camouflaged or mounted outside the production volume to provide a “clean” tank for the fish with few disturbances. While this study outlines the design and features of a specific system developed for fish experiments, the overall objective of this work is to introduce the idea to the research community that technological enhancements of research facilities can help achieve results that are more industrially relevant also under controlled laboratory conditions.

## 2 Materials and equipment

### 2.1 Experimental tank design

**FIGURE 1:**
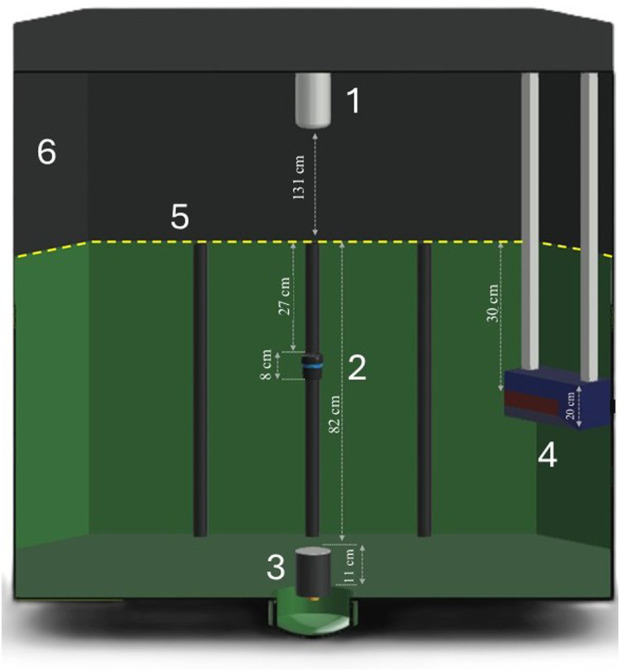
3D design of the tank environment featuring the top camera (1), a Ping360 sonar (2), an event camera (3), a stereo camera (4), LED lights (5) and a black tarpaulin (6).

The cyber-enchanced tank was built around a conventional indoor square fish tank (dark green colored) with dimensions 2 × 2 × 1 m, with a volume of approximately 4 $m^{3}$ . Figure 1 shows the 3D design of the tank, demonstrating the placement of the lights and instruments comprising the sensor suite.

#### 2.1.1 Illumination

**FIGURE 2:**
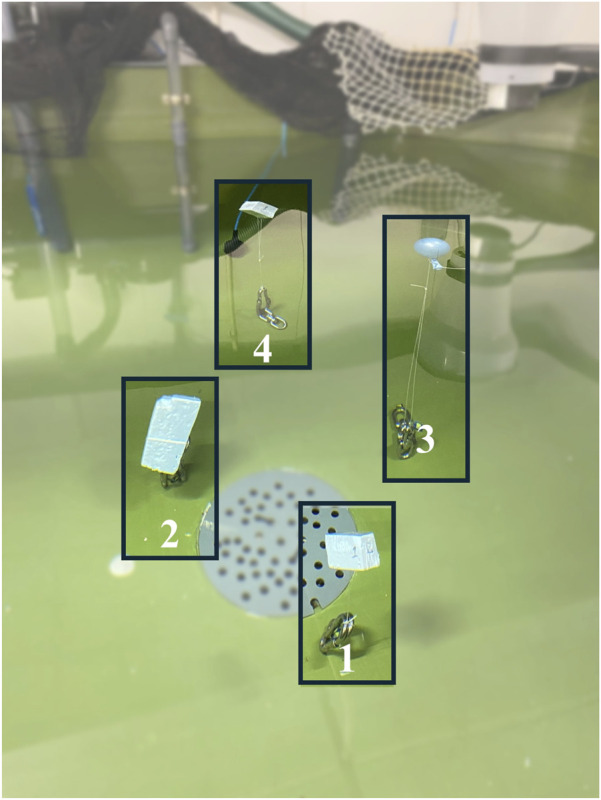
Sonar deployment based on four dummy objects representation.

**FIGURE 3:**
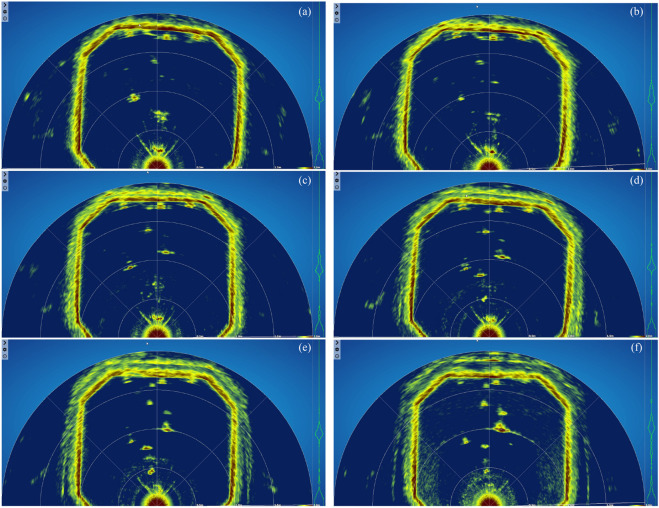
Dummy objects observed by the sonar when placed at various depths. Images were obtained using the PingViewer software. **(a)** 20 cm depth **(b)** 30 cm depth **(c)** 40 cm depth **(d)** 50 cm depth **(e)** 60 cm depth **(f)** 70 cm depth.

**FIGURE 4:**
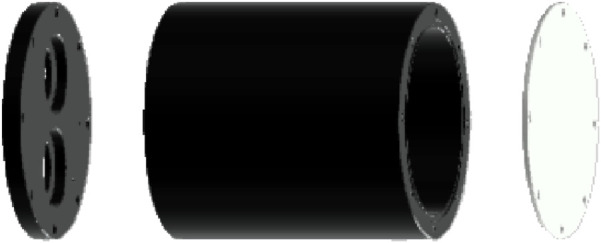
3D exploded view of the submersible event camera housing (attaching elements nor O-rings not shown).

**FIGURE 5:**
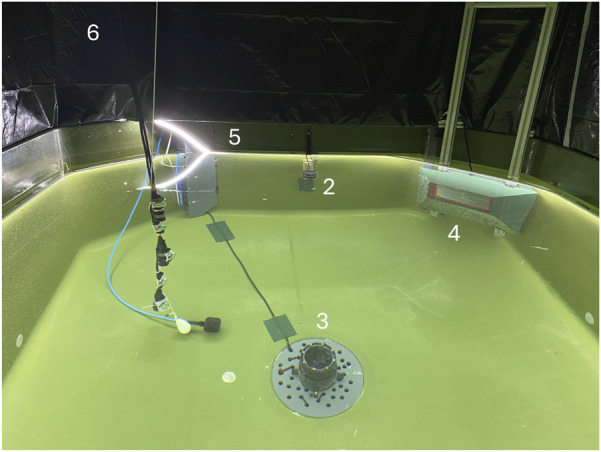
The actual experimental tank environment used in this study showing the Ping360 sonar (2), the event camera (3), the stereo camera (4), the LED lights (5) and the black tarpaulin (6) deployed in their respective positions. The topside surveillance camera is not shown as it was mounted in the ceiling of the room and was hence not captured in this image.

**FIGURE 6:**
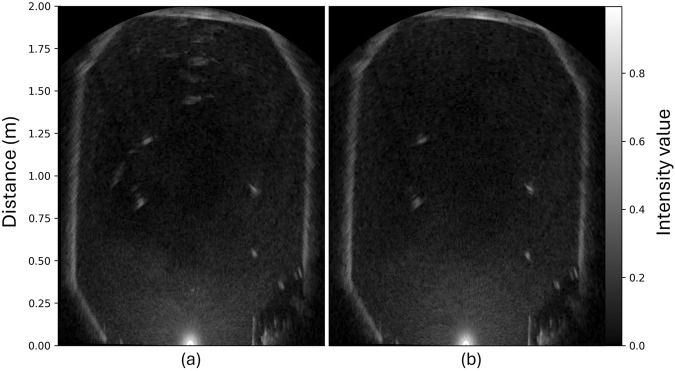
Example outputs from the scanning imaging sonar during preliminary fish trials in the CET. **(a)** depicts fish in a non-agitated state while **(b)** the output when the fish responded to an acute disturbance and sought toward the bottom and hence out of the observation volume. The four white blobs present in both images are detections of static sensors deployed in the tank volume.

The main aim of the illumination system was to simulate the light spectrum in commercial fish farms, and thus provide light conditions that resemble those experienced by farmed fish under production. This was performed by a submersible monochrome LED line (LED Neon S F22B-BH, Nortronic AS) attached to a specially designed support frame placed along the inner tank perimeter approximately 5 cm below the water surface, providing homogeneous “sunlight” illumination with a color temperature of 5600K–6500 K. These particular LEDs were chosen due to their color rendering index (CRI) of 80 which ensures good underwater visibility of objects, and their IP68 waterproof rating which would ensure safe submerged operations. The support frame stabilized the LED strip line at a fixed vertical position, providing illumination of the whole tank due to the wide opening angle of the LED strip at 112.3 ° . Moreover, this placement of the lights also prevented potential collisions between fish and lights, which could have been a welfare risk during trials. Also, a DALI LED driver (24 V DC-240 W) could generate a signal that would control the LED lights in their rated voltage range. To prevent other external light sources from perturbing the lighting conditions in the tank, a black tarpaulin was extended from the upper edge of the tank to the ceiling (Figures 1–6).

#### 2.1.2 Tank color and camouflage measures

To stimulate positive welfare in the fish, the tank was colored a dark green believed to not adversely impact salmon behavior or welfare. This also provided a dark backdrop for the fish that resembles the color of deep water beneath a fish farm when perceived from within a commercial cage. Furthermore, to ensure a clean environment for the fish, all sensing technologies and inlet pipes were concealed from the fish when possible. The intention of this measure was to reduce the potential impact of external factors on the fish, thereby facilitating an environment better suited for experiments targeting responses to specific stimuli.

### 2.2 Sensor suite

The sensor suite featured: 1) a scanning sonar (Ping 360, BlueRobotics Inc.); 2) a event camera (DAVIS 346, iniVation AG, Switzerland); 3) a stereo video setup (Alvium 1500 C-510 NIR, Allied Vision GmbH); and 4) a surveillance camera mounted in the ceiling above the tank (Reolink RLC 823a, Reolink). This provided a multi-modal observation method that enables a more detailed and deeper insight into fish behavior during tank trials (see Appendix Table A1 for sensor suite costs).

#### 2.2.1 Sonar system

The acoustic scanning sonar used in the sensor suite was a Ping360 scanning imaging sensor (BlueRobotics Inc., USA) which is a low cost imaging sonar that was first created for navigation and imaging purposes when deployed on underwater vehicles. This system has the ability to operate at great depths and can be controlled via open source software. The Ping360 emits a single acoustic beam that is mechanically moved across sector of angles, thereby stepwise assessing the backscattering for each angle in that sector. These values are then compiled into a 2D image describing the backscattering received across the range of angles and across the beam range (i.e., the maximum distance the Ping360 is set to measure). This results in an image not unlike those obtained using much more costly multibeam systems (e.g., Kristmundsson et al., 2023), but at a cost of lower update frequency (i.e., a full 360 ° scan using a Ping360 takes at least 8 s). Although previous studies have used the Ping360 to study fish in sea-cages (Zhang et al., 2024), the present study is, to our knowledge, the first case where this or similar tools have been tested in small land-based tank facilities designed for controlled fish trials. Based on the system specifications (Table 1) and the mechanical scanning ability of the sonar, it was placed at the tank wall facing in toward the tank center (Table 2) and set to scan 180 ° as this would be sufficient to capture an entire tank cross section (Figures 1, 2).

**TABLE 1:** Technical specifications of the Ping360 sonar.

Parameters	Values
Power supply (DC)	11–18 V (5 W)
Beamwidth (Horizontal)	2 °
Beamwidth (Vertical)	25 °
Minimum range	0.75 m
Maximum range	50 m

**TABLE 2:** Sonar setup.

Parameters	Values
Scanning range (m)	2
Receiver gain	Low
Sector angle ( ° )	180
Transmit duration ( μ s)	5
Transmit frequency (kHz)	1000
Speed of Sound ( $ms^{-1}$ )	1500

To identify the best sonar placement depth for capturing and visualizing fish shoals within the tank volume, a series of measurements were taken and analyzed using the PingViewer software (BlueRobotics). In these measurements, four dummy objects were placed at known distances (Figure 2) from the frontal side of the tank that were considered representative of real fish positions when the tank is stocked, while the sonar position was varied between 20 and 70 cm (Figure 3). The objects were placed approximately 0.7 m (object 1), 0.9 m (object 2), 1.1 m (object 3) and 1.4 m (object 4) from the sonar, respectively.

The results of this trial showed that a sonar placement between 30 and 40 cm (Figures 2, 3) gave a better visualization of the objects than the other depths, thus the sonar was fixed at a depth of 35 cm.

Based on a previous study (Hasan et al., 2024), in which a similar approach was implemented and knowing the operational principles mentioned by BlueRobotics, the sonar was placed with a small inclination of 10 ° relative to the horizontal plane. The main goal of doing this was to avoid potential surface reflections and thereby improve the data quality. Maximum scanning range was set to 2 m since this is sufficient to cover the entire tank volume. The limited volume also prompted setting receiver gain to “low”, since this may limit the impact and duration of multipathing and reverberation, and since we consider unnecessary a high gain considering the short distances achieved in the tank. The parameters describing the transmission pulses, i.e., transmit duration and pulse frequency, were set to 5 μ s and 1000 kHz, respectively. Finally, the speed of sound parameter in the Ping360 was set to 1500 $ms^{-1}$ which is an approximate value for saltwater.

Although the PingViewer software is useful for briefly reviewing sonar scans, we programmed a software pipeline for processing and interpreting the sonar signal, allowing us the use of more advanced processing methods beyond visualization of the data.

#### 2.2.2 Event vision

A Event Vision (EV) is a relatively recent technology type that detects changes in the brightness of individual pixels and registers these as events, which is a different approach than that used by traditional optical cameras. Instead of acquiring visual data as images each consisting of the full set of pixels that seek to match the observed motif, a EV operates by continuously monitoring pixel states. If the brightness detected by a pixel changes more than a specified threshold value, the EV will register this as an event. The output from the device at each time step is the total set of events since last time step, i.e., the pixels whose changes in brightness exceeded the threshold value. In consequence, the EV will continuously return the location and intensity of events when observing a dynamic scene. Conversely, a EV observing a static scene will output zero events and hence generate no data. This feature is particularly well suited to detecting motions in the images and results as the EV needs a much smaller amount of data to describe movements than a conventional camera. In addition, as it is not reliant on entire frames that are registered in cameras, the EV can operate at much higher speeds, registering events with a time steps of 1μs . Some recent studies used event-based cameras in cars and drones (Gallego et al., 2022; Gallego and Scaramuzza, 2017; Rebecq et al., 2019). However, there are very few, if any, examples of experimental case studies exploring their application in animal production on land or in water, and to our knowledge, there exist no previous studies using event camera technology in aquaculture. The intention of choosing this technology for the cyber-enhanced tank (CET) was to enable the use of registered events as direct indicators of movements and changes in motion patterns exhibited by fish, thereby avoiding the need for heavy and time-consuming video analysis algorithms as would be the case with conventional camera technology.

We used a DAVIS 346 event camera (iniVation AG, Switzerland) (IniVation, 2020) in the sensor suite (see Table 3 for specifications). To operate this system in an aquatic environment, we developed a specially designed submersible housing (Figure 4) to protect the camera against water leakage and maintain the system integrity in such challenging conditions. The housing consisted of a main cylindrical protective frame (made from POM-C Polyoxymethylene), sealed at the ends by two plates (made from polymethyl methacrylate), one of which was transparent. This protective enclosure enabled a more secure approach and at the same time quality data acquisition. The housing was designed to integrate properly with the environment of the tank in providing a “clean” arena for the fish without sharp edges. Moreover, there were no leakages during testing and preliminary experiments. As depicted in Figure 1 (marked by 3), the event camera can be placed at the bottom of the tank, projecting an upward field of view. Alternatively, the camera can be placed at the top of the tank (close to the surveillance camera marked by 1 in Figure 1), capturing a different view of the dynamics of the tank environment. Which of these positions to be chosen depends on the aims of the experiment. For instance, while bottom placement may be best to capture minute details on individual responses, topside mounting may perform better at identifying shoal-level responses.

**TABLE 3:** Technical specifications of DAVIS 346 event camera.

Parameters	Values
Dimensions (mm)	H 40 × W 60 × D 25
Spatial resolution	346 × 260 pixel
Camera dynamic range	55 dB
Maximum range	50 m
Temporal resolution	1 μ s
Event dynamic range	120 dB

#### 2.2.3 Conventional cameras

While the scanning sonar and the event camera provided the cyber-enhanced tank with observation modes previously untested in tanks designed for controlled fish experiments, the tank was also equipped with two conventional camera systems. This is useful for fish experiments in both enabling the validation of the new observation methods and resulting in a more robust total package for fish observation. To enable overview images covering the entire tank, a high-resolution surveillance camera (Table 4) was mounted on top of the tank (Figure 1).

**TABLE 4:** Technical specifications of the top camera.

Parameters	Values
Sensor type	Reolink RLC 823A
Spatial resolution	3840 × 2160 pixel
Sensor type	CMOS
Sensor size	Type 1/2.8
Max. frame rate	25 FPS
Night vision	Four IR LEDs

To also get a sideways view of the fish and enable positional tracking, a stereoscopic vision camera was mounted on one of the tank sides at a 30 cm depth facing inwards toward the tank center (Figures 1–4). The stereo camera consisted of two Alvium 1500 C-510 NIR cameras (Allied vision), with a resolution of 2592 × 1944 pixel and that had a horizontal, vertical and diagonal field of view of 41.2, 26.8 and 63.6 mm, respectively (Table 5). As depicted in Figure 1 (1, 4), the two camera systems thus enabled viewing a fish shoal from above and from the side.

**TABLE 5:** Technical specifications of the stereo camera.

Parameters	Values
Sensor type	Alvium 1500 C-510 NIR
Spatia resolution	2592 × 1944 pixel
Sensor type	CMOS
Sensor size	Type 1/2.5
Pixel size	2.2 μ m × 2.2 μ m
Max. frame rate	68 FPS
Lens	C Series VIS-NIR
Focal length	3.50 mm
Field of View	Horizontal: 41.2 mm - 102.4
	Vertical: 26.8 mm - 82.3
	Diagonal: 63.6 mm - 117

### 2.3 Control unit

To ensure steady operation during data capture, a mini computer built around a Jetson AGX Orin (NVIDIA Corp.) with high performance skills (Table 6) was used. The main role of this system was to manage the two novel sensor technologies, (i.e., the event camera and the sonar), and to avoid synchronization issues the operating code was written using multithreading python.

**TABLE 6:** Technical specifications of NVIDIA Orin AGX.

Parameters	Values
Memory	64 GB 256-bit LPDDR5
Storage	64 GB eMMC (Internal)
	1 TB (External)
Cores	2048 NVIDIA CUDA
	64 Tensor

For each recording, the operative system first set the initial parameters and recording time and then started the multithreading data collection process. This prompted the event camera to collect the event and frame acquisition datasets with timestamps, while the sonar system did likewise with sonar scans. The operative system then stored both datasets in a designated folder. Since the systems were operated by the Jetson, the resulting datasets were synchronized.

## 3 Methods

The cyber-enhanced tank can contribute to better insight into and understanding of fish responses to situations and events in aquaculture production, as outlined in the following.

### 3.1 Illumination

The main advantage of the LED based illumination system in experiments lies in its ability to provide light that resembles that in a fish farm both in terms of intensity and color, and how it disperses in the tank volume. This will be important in trials seeking to study how farmed fish respond to specific events or situations arising under production as it will reduce the chance that responses to the illumination confuses the outcomes of the trial. Moreover, the ability to active control the light level with a high accuracy allows for replication of natural photoperiods. Since the cyber-enhanced tank is shielded from the impact of other artificial light sources, this allows for actively steering the light levels in the tank to follow any chosen photoperiod pattern. If further combined with active control of the temperature of the inlet water, the tank could thus be set up to actively mimic the conditions in a fish farm at any season irrespective of the actual time of year. Considering that seasonal variations may have direct impacts on farmed fish (e.g., Bowden et al., 2007; Versteeg et al., 2021) and hence how they respond to other stimuli, this could open for a more efficient experimentation where seasonal variation can be taken into account without requiring the experiments to span across actual seasons.

### 3.2 Sonar system

The most apparent use of the Ping360 sonar in describing fish behaviors and distributions is to use the data to visualize the spatial distribution of the fish. While this can give some insight into the spatial distribution of the fish, this system is generally not able to give insights into individual fish behaviors due to the relatively long scan time. However, recent studies using the same sonar type have demonstrated how more advanced processing of the data could be used to get added value from the Ping360 data, and several of these could also have potential uses in the cyber-enhanced tank concept. In a recent example from aquaculture, a deep learning based method was developed and applied to observe the distance farmed salmon preferred to keep from an intrusive object with various physical features (Zhang et al., 2024). Although this study was conducted in a full-scale sea-cage and hence at a much larger spatial scale, a similar approach could be adapted for tank use to provide measures of the distances to and sizes of fish shoals observed with the sonar. Other recent studies have sought to develop AI-based methods for more accurate object detection using the Ping360 sonar. This has for example, entailed the use of a U-net based segmentation model (Hasan et al., 2024) and applying the promptable Segment Anything Model (SAM) (Tolie et al., 2024) to segment and detect various objects observed by the sonar. While neither of these two latter cases targeted fish, these or similar methods are likely transferrable to the cyber-enhanced tank application as long as they can be adjusted to take the short observation range and low power settings into account. If properly trained on relevant data, segmentation methods could ultimately be used to count and even size the individual fish observed by the sonar.

### 3.3 Event vision

Since event cameras have never been applied in previous fish studies, there exist no examples of processing methods designed for deriving metrics relevant for analyzing fish behaviors. However, the direct analysis of the raw event output may in itself be valuable for describing the dynamics in fish shoals. Simple time series plots of the number of events in the image could as such be a good enough indicator for detecting the onset and scale of the behavioral response of fish when subjected to both acute and chronic factors. This approach could potentially be expanded to provide spatial indications on where in the image frame the activity occurs by dividing the image frame into a grid of cells and then counting the events within each cell. Such an approach could, for instance, shed light on variations in spatial distribution dynamics when the device is in topside position. The use of event cameras in other application areas led to the development of more advanced processing methods for further refining the outputs from event cameras, some of which may be relevant for describing fish. Examples of such methods include self-supervised frameworks that estimate optical flow based on event streams (Zhu et al., 2019), methods for image reconstruction based on event data (Scheerlinck et al., 2020) and real-time event-based stereo-visual odometry (Zhou et al., 2021), and approaches for fine grained object detection using event streams (Kim et al., 2021). These are just a few examples of methods emerging from the ongoing surge of studies that seek to use this technology, and several of these and other approaches may be adaptable to a fish experimental situation.

### 3.4 Surveillance and stereo cameras

Unlike the event camera technology, the conventional cameras used for surveillance and stereo video have been a staple within fish monitoring for decades. Consequently, there exist several methods designed to derive information on fish behaviors by processing camera footage. For instance, subjecting the video stream from the surveillance camera to methods such as optical flow (Beauchemin and Barron, 1995), surface activity using Convolutional Neural Networks (Ubina et al., 2021) or entropy and fractal techniques (Eguiraun and Martinez, 2023; Eguiraun et al., 2018; Eguiraun et al., 2014) can result in data describing the dynamics in the entire fish group. Likewise, stereo camera images can be analyzed using state-of-the-art stereo processing methods, many of which have been adapted to fish (Li et al., 2024b). Such adaptations have enabled the detection and tracking of, e.g., individual movements and behavior (Saad et al., 2024), wounds and defects (Nissen et al., 2024), and fish size (Silva et al., 2024). These are but a few examples from the expanding toolbox for analyzing video footage. The inclusion of these and similar methods in the processing of the output from the sensor suite will contribute to making the cyber-enhanced tank more diverse and robust in terms of observation modes. Moreover, since these established methods are usually validated, they can be used to generate ground truth data for validating new processing methods for processing the data generated by the event cameras and the sonar.

### 3.5 Sensor fusion approaches

The main motivation for equipping the cyber-enhanced tank with a diverse set of sensors was to enable multi-modal observation. While separately analyzing the data collected by each instrument is valuable in itself, the value can be multiplied if the data were to be merged into one holistic dataset describing the biological situation in the tank.

A first step on this pathway could be to look into combining the outputs from the vision based methods. Since the three methods used here describe different features of the observed fish, a combined view could potentially exploit the advantages of all methods. For instance, the surveillance camera will provide good inputs on the horizontal fish distribution and general shoal movements. Moreover, the fast sampling rate of the event camera compared with the other optical methods renders it much less sensitive to motion blur and other disturbances, meaning that the inclusion of event data in an analysis could contribute to better detection of details.

A drawback of all the vision based methods included in the sensor suite is that they require light to capture objects and events. This means that neither of these systems are likely to provide data when there is low natural or artificial light. Since the cyber-enhanced tank is intended to simulate the conditions in a fish farm including the natural photoperiod, the fish will at times be kept in darkness, especially when simulating night time conditions during winter. At such times, the optical methods will struggle to provide data on what the fish are doing. While this could, to a certain extent, be compensated with additional artificial lights. The inclusion of such lights may compromise the aim of keeping conditions as similar to a production situation as possible (unless the simulated case features artificial lights). In the cyber-enhanced tank concept, monitoring in darker periods is therefore intended to be facilitated by the Ping360 sonar. Although the sonar by itself can provide insight into behaviors during darkness, added value can be obtained by fusing the sonar output with the visual data. While such an integration may not be as seamless as when merging the three optical methods, the co-analysis of sonar data with video would have a dual purpose. Firstly, it would make the sensor suite more robust in providing yet another mode of observation. Secondly, the data collected during brighter periods when all systems are running can be used to validate the sonar output and develop methods for analyzing the resulting data to gain deeper insights into fish responses.

Finally, a long term aim will be to integrate digital twin technology (Rasheed et al., 2020) in the cyber-enhanced tank concept. This will require integrating the sensor suite with mathematical models describing the system dynamics, either in the form of Knowledge Based Models (KBM) synthesizing existing system knowledge into mathematics or Data Driven Models (DDM) that can predict response patterns based on a set of input variables (Føre et al., 2024).

## 4 Results

This chapter demonstrates the outcomes of the novel tank setup developed in this study, thus illustrating the first iteration of the cyber-enhanced tank concept for fish experiments. Figure 5 displays an image of the experimental tank with the sensor suite. As depicted, the submersible LED line (Figure 5) is immersed below the surface along the edge of the tank, while the sonar is installed at the farthest wall (Figures 2–5). The stereo camera (4) is placed at the same depth as the sonar, while the event camera in its waterproof housing/frame (in this case) is placed at the bottom of the tank (3). Although not visible in the image, the surveillance camera was mounted directly above the center point of the tank facing downwards.

### 4.1 Experimental fish and ethical permit

The data used to demonstrate the systems were collected during preliminary trials before an upcoming stress experiment using the CET that was in compliance with the Norwegian animal welfare act under approval by the Norwegian Animal Research Authority (permit no. 30968). The full trials lasted from January to March 2025, during which the CET contatined a maximum of 150 Atlantic salmon (*Salmo salar* L.) post-smolts. The average length and weight of the fish were 25.56 ± 1.46 cm and 164.80 ± 25.50 gr, respectively.

### 4.2 Sonar

Figure 6 presents an image obtained by the sonar during preliminary trials demonstrating the dynamics of a fish group in the tank volume and the tank wall are visualized. The Ping360 registers the strength of the reflected signal from each object appearing in the beam. These images were obtained using Python code developed specifically for processing the sonar data collected from the cyber-enhanced tank that mapped intensity values to values between 0 (black = low intensity) and 1 (white = high intensity) in the figure. The location of the sonar is represented as a white glowing half-circle shape at the lower edge of the image, while the tank wall, which reflects high intensity, is rendered as a white contour. White patches (Figure 6a) within the tank boundaries are objects in the tank volume, in this case, a fish shoal that is clustered at the side of the tank opposite to the sonar. Conversely, Figure 6b depicts a situation where the shoal had been startled by a sudden event that caused them to move away from the observation area.

### 4.3 Event camera

**FIGURE 7:**
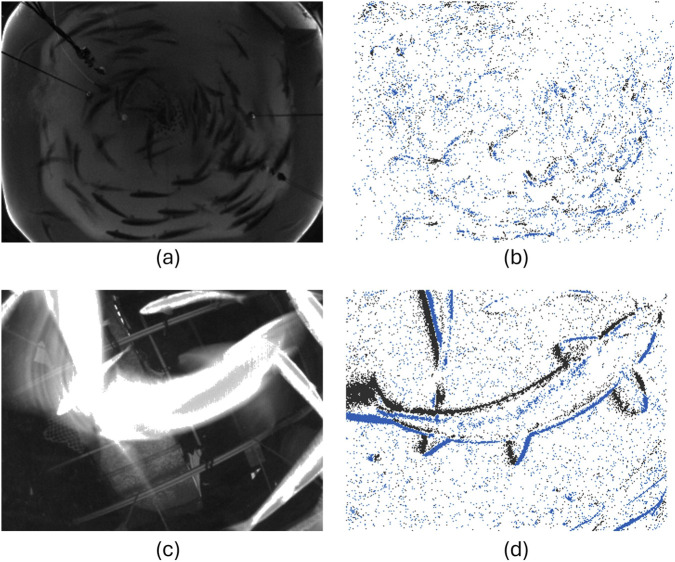
Example output from the event camera in the CET during feeding in preliminary fish trials from top **(a,b)** and bottom perspectives **(c,d)**, with monochrome frames (left) and event images (right).

In being a completely novel way of observing fish, the outputs from the event camera can best be described when compared with the outputs from conventional cameras. Since the event camera used in the present version of the cyber-enhanced tank can provide regular monochrome images in addition to events, it is also reasonable to compare monochrome image outputs from a motive with the corresponding events output. Two example outputs obtained during feeding events in the preliminary trials are presented in Figure 7, where conventional monochrome image frames are placed on the left (a and c) and corresponding event images are provided to the right (b and d). The image pair on the top of the figure was collected with the event camera placed topside (i.e., next to the surveillance camera), while the lower image pair was collected when the device was mounted at the bottom. Blue and black dots in the event images (b and d) indicate a decrease or an increase of the light intensity in a pixel as registered by the event camera. These examples clearly show how static details and features that are present in the regular images are not picked up by the events, while dynamic details such as movement are clearly detected as events. The two camera placements offer different insights into how the event camera can be used in that the topside position gives data on the entire tank while the bottom position in not enabling a full view of the tank, gives data on fewer fish but at a much closer distance.

Moreover, the images also show how the events are mostly concentrated around the fish, which are the most mobile objects in the image. Another detail that is apparent from these images is that event outputs from the device are practically insensitive to motion blur which can often be a major challenge in computer vision applications with conventional cameras. This is particularly clearly seen in the images taken from the bottom position in that these capture a fast moving fish at a close distance, both of which are factors that tend to increase the challenge of motion blur. While the outline of the fish is blurred in the monochrome image (left), it is sharp and clear in the event image (right). This illustrates the potential of this technology in visualizing fish even in situations where they are indistinguishable in regular images.

**FIGURE 8:**
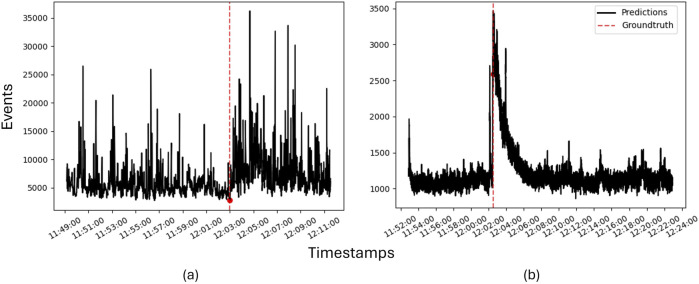
Time series of the summed up number of events registered before and during feeding with **(a)** the event camera mounted on the tank bottom and **(b)** the event camera mounted topside.

To provide a more concrete example of quantitative use of the events, Figure 8 shows two time series of the summed up events (i.e., number of black and blue dots in the event images) during feeding trials. The feeding time is marked by the red dotted line. While it is clear that both camera positions imply the system was able to detect the onset of feeding, bottom deployment (Figure 8a) gave generally higher number of events but more variations as would be expected since it is more sensitive to individual fish entering and leaving the observation volume. Conversely, when the system was mounted topside (Figure 8b), the number of events was more homogeneous before and after the onset of feeding, likely because it captured the entire shoal and thus was less sensitive to individual fish variations. The lower total number of events is because the fish were further away from the camera and thus represented fewer pixels.

### 4.4 Conventional cameras

**FIGURE 9:**
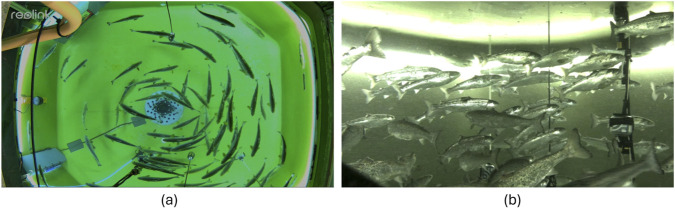
Outputs from the conventional camera systems when the fish were in a non-agitated state in the CET showing outputs from **(a)** the ceiling mounted surveillance camera and **(b)** the side mounted stereo camera.

To illustrate the raw outputs from the surveillance and stereo cameras mounted in the tank, Figure 9 presents an overview image of the experimental tank from perspective of the surveillance camera (a), and a side view of the fish collected by one of the stereo cameras (b). Both images were collected during preliminary trials in the CET and show a group of fish in a non-agitated state where the fish exploit much of the tank volume.

## 5 Discussion

The emergence of new technologies, many based on biologically/nature inspired approaches (e.g., genetic algorithms and artificial intelligence), are opening new avenues for fish monitoring. However, it is important not only to develop and systematically test these technologies in laboratory setups but also to test them in environments that resembles the real farming situation as closely as possible.

### 5.1 Providing a realistic tank environment

The cyber-enhanced tank concept outlined in this study aspires to provide an environment that simulates the light conditions that prevail in fish farming units, while offering the fish a “clean” environment where sensors and other components are concealed. In combining this with the collection of comprehensive and varied data sets describing the fish responses, the setup is well suited for analyzing fish responses to tank management. To our knowledge, this is one of the first tank based system seeking to imitate conditions in an aquaculture unit while also retaining the main advantages of a small and highly controllable experimental setup.

### 5.2 Sensor suite

#### 5.2.1 System performance and comparison of sensing modes

The sensor suite applied in this study represents the first case reported in literature (to our knowledge) where event data interpretation is used to analyze fish movements. One of the most attractive features of using event cameras is the reduced need for post processing to detect motions compared with conventional cameras. This means that an event camera, given the proper post processing algorithm, should require much lower processing effort than conventional vision cameras. The preliminary analysis methods presented here use basic measures such as identifying events related to fish movements and did find these to be linked with fish movements. A particularly promising outcome from the resulting data was that the event camera appeared able to capture the clear outlines of fish moving fast enough to induce motion blur in conventional images. This harmonizes with observations made when analyzing the use of event cameras in other segments (Chakravarthi et al., 2024). This is because events are registered at a much higher rate ( 1μs time interval) than conventional cameras ( $\frac{1}{30}s$ time interval with 30 fps). While it could be argued that the same could be achieved by using a high speed camera, systems capable of frame rates well beyond 30 fps tend to be expensive. Moreover, studying the sheer quantity of events over time demonstrated that the event camera was able to detect the differences in behavior for feeding and non-feeding fish, thereby demonstrating the utility of this system in quantifying such responses. From looking at the event outcomes in more detail (Figure 7 and ??), the nature of the response and the response time are inextricably linked to the system deployment. While both topside and bottom positions allowed monitoring the spatial response of the fish to the feeding procedure, it appears that topside mounting detected a response at an earlier time and hence closer to the actual onset of feeding than when the device was placed at the bottom. This is linked with that top deployment captured the whole group of fish while bottom deployment gave a much smaller field of view where only a small group of fish could be monitored at any given time. In turn, this implies that topside mounting is better for detecting the specific onset of a group response in the fish, but that bottom mounting allows the collection of data more connected with the behaviors of the individual fish. While the metrics used in this study are promising and can be used to gain information on both shoals and individual fish, future development should explore the potential of subjecting the data to more advanced processing methods.

A combination of events and conventional outputs (such as stereo video) could yield the movement velocities of individuals. Since the sum of individual movements is what causes the shoal movements observed by the surveillance camera, this setup would enable two different modes to observe the same phenomenon. Moreover, the stereo system could also provide individual fish sizes and size distribution, while surveillance camera data could be processed to assess the number of individuals, in sum providing a biomass estimate. These two systems could then be complemented by the event camera, which is much better equipped to accurate and rapid detection of movements than the former two systems. Since the event camera is expected to detect dynamical changes faster than other cameras, the event camera could provide early warnings when something is inducing behavioral changes in the fish. In any case, the utilization of event cameras in aquaculture will introduce a new method of observing and imaging animals in aquatic environments. The advantages of such case study underscore the importance of novel methodologies that offer less power consumption with increased efficiency.

The main idea behind including the Ping360 sonar in the suite was to enable observation when light is insufficient for the optical sensors. By compiling the outputs from the Ping360 sonar into a 2D-image, it is possible to observe both the fish and their shoaling behavior relative to the tank edges. In the preliminary analyses of these data, the 2D-images were simply used to detect the presence or absence of fish. However, more complex methods are possible to apply to the resulting data to acquire other and more specific metrics related to the fish, as previously done in sea-cages (Zhang et al., 2024).

Although the event camera and the Ping360 sonar represented the most novel elements, the addition of a surveillance camera and stereo cameras enabled a more robust sensor suite. The additional observation of the tank through these vision sensors provide a different view of the fish in the tank. Data from these can also be used to validate the event camera in future studies, to provide overview topside images of shoal behaviors and images of individual movements from the side. Glare and reflections due to external lights may mask and distort the fish and cause challenges for the analysis of conventional images if not properly handled. The lighting setup in the cyber-enhanced tank concept contributes to improving this as its underwater placement results in no reflections, as evidenced by the images captured with the camera systems in this study.

#### 5.2.2 System limitations

All technological advancements will face operational difficulties when applied in new environmental contexts and new applications. This also applies to the current study although measures such as redundancy in sensor systems and actively designing and partially controlling the tank environment. As a whole, the introduction of the multi-modal sensor suite increases the chances of success during difficult conditions (e.g., with low light, low fish mobility or high turbidity) as the collaborative work of the systems can overcome the respective challenges of each sensor system. However, to map the full capabilities of the sensor suite, it is necessary to explore the limits of each of the subsystems separately. Conventional cameras (i.e., the surveillance and stereo cameras) are designed to perform best in ideal visibility conditions with, e.g., optimal lighting and low turbidity, but their image quality will gradually deteriorate when conditions worsen, as will the potential of obtaining quantifiable data from their analysis. The event camera will be less sensitive to reduced illumination since it produces events due to changes in pixel brightness directly and does not need for the motive to be clear enough for the application of methods such as object detection and segmentation to provide data. While this reduced need for post processing is one of the main perks of using event cameras instead of conventional cameras, it can also make these systems more sensitive to other features such as turbidity. This is because they, in relying on pixel changes, do not distinguish between pixels pertaining to a fish and pixels that are not associated with the fish. In turn, this means that the movement of particles and other objects will be equally expressed in the event data as fish motions, which in cases where turbidity is high or there are other fish (e.g., cleaner fish, wild fish) or moving objects in the cage can cause challenges in isolating the fish responses. Despite the different advantages of the optical methods in the suite, neither of these will produce data in darkness as they are inherently based on the reception of light. This is partly solved by the complementary data obtained with the Ping360 sonar which is not significantly affected by the absence of lighting or high turbidity. However, while this means that the fish can be observed through the sonar data when the other systems are not able to provide data, the Ping360 operates at a much lower sampling rate ( s between samples) than the conventional ( ms between samples) and event ( μs between samples) cameras and will hence not be able to provide as dynamic and high resolution outputs as these.

### 5.3 Concluding remarks

In conclusion, this study has introduced a new concept for how future experiments on farmed fish can be conducted in a highly controlled tank environment while still featuring conditions similar to those experienced in commercial farms. The resulting cyber-enhanced tank concept features a multi-modal sensor suite can provide deeper insight into fish responses. This will be practical when environmental conditions are challenging for automated observation due to factors such as low light intensities, high turbulence and high turbidity. The next steps in this research are to use this first version of the cyber-enhanced tank setup in experiments where detailed data is collected from the different sensors and processed to detect fish movement patterns when they are subjected to various external effects such as stressors. This will both demonstrate the utility of this concept for future use in fish experiments that aim to provide industry relevant knowledge while still being controllable, and provide necessary experiences and inputs on how the concept can be refined further to fulfill its intended role in the scientific toolbox. Being this the first study using event camera technology to observe fish responses, that will also aspire to validate and explore the utility of event cameras in detecting stress responses in fish. The resulting data will also provide a foundation for developing new methods for processing data from the sensors both separately and in combined multi-modal analyses.

## Data Availability

The raw data supporting the conclusions of this article will be made available by the authors, without undue reservation.

## Appendix

### Equipment costs

Table A1 summarizes the costs of all the main components used in the setup of the tank and the sensor suite.

**TABLE A1:** Costs of each individual sensor comprising the sensor suite.

Sensor suite	Cost (EUR)
Event camera (DAVIS 346)	3,900
Sonar (Ping 360)	2,412
Top camera (Reolink RLC 823a)	275
Stereo camera (Alvium 1500 C-510)	910
Submersible lights (LED Neon S F22B-BH)	1,780
Jetson AGX Orin	1,410
Total	10,687
